# Letter to editor concerning article titled “The negative pressure wound therapy may salvage the infected mesh following open incisional hernia repair”

**DOI:** 10.1016/j.amsu.2021.102191

**Published:** 2021-03-06

**Authors:** Rohit Gupta, Ashesh Kumar Jha, Manoj Kumar, Surya Vikram, Manoj Kumar

**Affiliations:** Department of General Surgery, All India Institute of Medical Sciences, Patna, 801507, India

**Keywords:** Mesh infection, Wound, Hernia, Negative pressure

Dear Editor,

We read with great interest the article by K. Boettge et al. [1] “The negative pressure wound therapy may salvage the infected mesh following open incisional hernia repair” published in Annals of Medicine and Surgery volume 61, pages 64–68. We would like to appreciate authors approach of utilizing negative suction wound therapy for mesh infections in incisional hernia. Authors have reported a retrospective study of 30 patients, undergoing negative suction wound therapy for mesh infection in incisional hernia from 2007 to June 2020. We agree with the fact that mesh infection is a serious complication and difficult to manage, eventually leading to removal of the mesh. In such a scenario negative suction wound therapy may add to surgeon's armamentarium to salvage the mesh. However, a peculiar observation in noted in Table 2 of the article [1]. Although authors have mentioned 13 patients underwent component separation by transversus abdominis release while sublay procedure [2] was performed in only 11 patients out of total number of 30 patients. Transversus abdominis release (TAR) includes posterior component separation, described as a myofascial release technique in surgery of complex ventral hernia [3]. In onlay technique as described by Chrevel [4] mesh is placed anterior to the defect and role of transversus abdominus release is questionable.

**Table 2:** Perioperative data I

Variable		Study group
		n = 30
Mesh placement	*onlay*	19
	*sublay*	11
Component separation^a^		13
Primary incisional hernia		18
Relapse		12
Operating time	*minutes*	122.6 (51.1)

Continuous measurements are presented as mean (SD).

a TAR transversus abdominis release.

We consider that this article has enlightened us about the use of negative suction therapy and its utility in salvaging mesh from infection. Additional description about component separation and technique employed will further enhance our understanding of the article and will be beneficial to all readers.

## References

[1] Boettge K., Azarhoush S., Fiebelkorn J., De Santo G., Aljedani N., Ortiz P., Anders S., Hünerbein M., Paasch C. (2020 Dec 23). The negative pressure wound therapy may salvage the infected mesh following open incisional hernia repair. Ann Med Surg (Lond).

[2] Rives J., Pire J.C., Flament J.B., Palot J.P., Body C. (1985). Le traitement des grandes éventrations. Nouvelles indications thérapeutiques à propos de 322 cas [Treatment of large eventrations. New therapeutic indications apropos of 322 cases]. Chirurgie.

[3] Novitsky Y.W., Elliott H.L., Orenstein S.B., Rosen M.J. (2012 Nov). Transversus abdominis muscle release: a novel approach to posterior component separation during complex abdominal wall reconstruction. Am. J. Surg..

[4] Chevrel J.P. (1979 Feb 24). Traitement des grandes éventrations médianes par plastie en paletot et prothèse [The treatment of large midline incisional hernias by "overcoat" plasty and prothesis (author's transl)]. Nouv. Presse Med..

